# Prediction of aromatase inhibitory activity using the efficient linear method (ELM)

**DOI:** 10.17179/excli2015-140

**Published:** 2015-03-20

**Authors:** Watshara Shoombuatong, Veda Prachayasittikul, Virapong Prachayasittikul, Chanin Nantasenamat

**Affiliations:** 1 Center of Data Mining and Biomedical Informatics, Faculty of Medical Technology, Mahidol University, Bangkok 10700, Thailand; 2 Department of Clinical Microbiology and Applied Technology, Faculty of Medical Technology, Mahidol University, Bangkok 10700, Thailand

**Keywords:** efficient linear method, genetic algorithm, aromatase, aromatase inhibitors, QSAR, data mining

## Abstract

Aromatase inhibition is an effective treatment strategy for breast cancer. Currently, several *in silico *methods have been developed for the prediction of aromatase inhibitors (AIs) using artificial neural network (ANN) or support vector machine (SVM). In spite of this, there are ample opportunities for further improvements by developing a simple and interpretable quantitative structure-activity relationship (QSAR) method. Herein, an efficient linear method (ELM) is proposed for constructing a highly predictive QSAR model containing a spontaneous feature importance estimator. Briefly, ELM is a linear-based model with optimal parameters derived from genetic algorithm. Results showed that the simple ELM method displayed robust performance with 10-fold cross-validation MCC values of 0.64 and 0.56 for steroidal and non-steroidal AIs, respectively. Comparative analyses with other machine learning methods (i.e. ANN, SVM and decision tree) were also performed. A thorough analysis of informative molecular descriptors for both steroidal and non-steroidal AIs provided insights into the mechanism of action of compounds. Our findings suggest that the shape and polarizability of compounds may govern the inhibitory activity of both steroidal and non-steroidal types whereas the terminal primary C(sp3) functional group and electronegativity may be required for non-steroidal AIs. The R code of the ELM method is available at http://dx.doi.org/10.6084/m9.figshare.1274030.

## Introduction

Cancers are important health issues due to their life-threatening consequences and impacts on the quality of life. Breast cancer is the most common cancer in women and is ranked as the second most common cause of death in women worldwide (Yeo et al., 2014[34]). The incidence of breast cancer has continuously increased despite improved diagnostic and surgical techniques (May, 2014[15]). Therefore, worthy attention has been drawn to the treatment and prevention of this cancer to improve the survival rates and quality of life. Estrogen is a steroidal hormone that is essential for many physiological functions (Couse and Korach, 1999[8]; Cutolo and Wilder, 2000[9]; Martín-Millán and Castañeda, 2013[14]; Michet Jr et al., 1985[16]; Pettersson and Gustafsson, 2001[22]; Straub, 2007[29]). However, estrogen can facilitate the growth of many estrogen-dependent cancers, including breast (Osborne, 1998[21]; Simpson et al., 2000[28]) and endometrial cancer (Watanabe et al., 1995[31]; Yamaki et al., 1985[32]; Yang et al., 2002[33]). The synthesis of estrogen is a multistep process in which the rate-limiting step of production is facilitated by the aromatase enzyme (Recanatini et al., 2002[23]). Thus, the inhibition of the aromatase enzyme leads to a decreased amount of estrogen products and is considered an effective treatment strategy for breast cancer (Brueggemeier et al., 2005[3]). Recently, many aromatase inhibitors have been developed and clinically used for breast cancer treatment with favorable treatment outcomes (Sainsbury, 2013[25]). Aromatase inhibitors are classified according to their chemical structure and mechanism of action into steroidal and non-steroidal types (Recanatini et al., 2002[23]). It should be noted that the inherent properties of each type might govern the interaction with the aromatase enzyme, rendering the inhibitory activity.

Computational approaches have become versatile tools in drug development. Recently, quantitative structure-activity relationship (QSAR) was utilized for predicting the aromatase inhibitory activity of steroidal and non-steroidal AIs using a decision tree method with acceptable prediction results (Nantasenamat et al., 2013[19]). Although useful and interpretable, the aforementioned model affords performance with significantly different values between the training and 10-fold cross-validation (10-fold CV) sets with accuracies of 92.22 % and 71.67 % for steroidal AIs as well as 93.88 % and 76.79 % for non-steroidal AIs. It can be assumed that either the molecular descriptors or the learning method (Nantasenamat et al., 2013[19]) was not optimal for predicting the activity of steroidal and non-steroidal AIs. Previously, support vector machine (SVM) had been successfully used to model a wide variety of biological activity. In fact, such SVM-based model is well recognized as one of the most powerful learning approach outperforming other learning methods such as artificial neural networks (ANN) and multiple linear regression (MLR) (Attar and Bulun, 2006[2]; Brueggemeier et al., 2005[3], 1990[4]). The limitation of this model is its low interpretability whereby prediction is performed in a black-box manner, i.e., practitioners may not gain insights into which molecular descriptors highly influenced the activity/inactivity of chemical compounds. 

To alleviate those problems, building a QSAR model should greatly concern the following: (i) develop a generalized QSAR model that is established from the efficient optimization approach; (ii) construct a QSAR model that can automatically identify informative features from a large pool of molecular descriptors for providing a better understanding of the mechanism of chemical compounds; and (iii) provide a white-box approach that is simple, user-friendly and afford acceptable prediction results. 

In this study, we propose an efficient linear method (ELM) that can be utilized for both estimating the feature importance and constructing the QSAR model. Particularly, the ELM method estimates informative features from their score usage. Consequently, the ELM model is constructed in a straightforward fashion by considering only the weighted-sum product and the threshold. Prediction results indicated that the proposed ELM method was comparable to that of the SVM-based method and yielded an outstanding performance when compared to ANN-based method. Remarkably, these results indicated that the selected molecular descriptors provided improvements over the previous study (Nantasenamat et al., 2013[19]). The molecular descriptor importance was analyzed to provide insights in correlating molecular descriptors with their aromatase inhibitory activity. Results from performance comparison demonstrated that the proposed ELM method is an efficient and effective learning approach for predicting the aromatase inhibitory activity for steroidal and non-steroidal AIs thereby improving upon the previous approach. Furthermore, the ELM method could be used to analyze other chemical compounds* a priori*.

## Materials and Methods

### Data set

A large dataset of compounds affording aromatase inhibitory activities was obtained from our previous compilation (Nantaseamat et al., 2013[19]). This set contained 973 non-redundant compounds in which 280 and 693 were steroidal and non-steroidal AIs, respectively. Removing the intermediate activity with pIC_50_ values in the range of 5 to 6 resulted in a final set of 180 steroids (81 active and 99 inactive) and 474 non-steroids (349 active and 125 inactive) as summarized in Table 1(Tab. 1).

Molecular descriptors were also obtained from the aforementioned study in which they were based on low-energy conformers computed at the semi-empirical AM1 level. The descriptors constituted two subsets: (i) quantum chemical and (ii) molecular descriptors. Briefly, the former subset was comprised of mean absolute charge (*Q*_m_), energy, dipole moment (*μ*), highest occupied molecular orbital (HOMO), lowest unoccupied molecular orbital (LUMO) and the energy gap of the HOMO and LUMO states (HOMO-LUMO). The latter set is made up of 3,224 molecular descriptors that were computed from Dragon version 5.5.

### Efficient Linear Model

**Figure 1:**
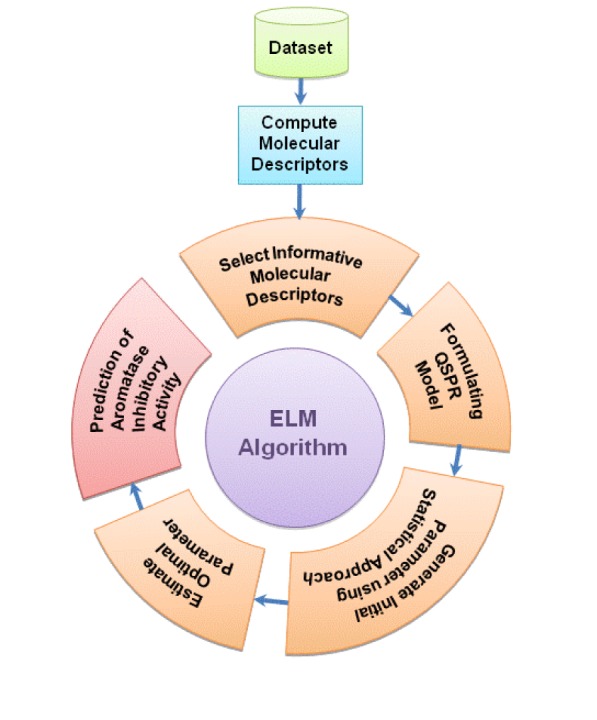
Workflow diagram of the efficient linear method (ELM)

The proposed ELM is a general-purpose method for establishing a QSAR model by identifying important descriptors that are well correlated with the activity. The predictive result was directly obtained from the weighted-sum product and threshold. A flowchart of the proposed ELM method is shown in Figure 1(Fig. 1) and its pseudocode is shown in Table 2(Tab. 2). The R code of the ELM algorithm is available at http://dx.doi.org/ 10.6084/m9.figshare.1274030. The procedure of the ELM method consists of the fol-lowing steps: (i) selecting informative molecular descriptors, (ii) calculating an initial parameter using a statistical approach, (iii) estimating an optimal parameter for enhancing the performance of the ELM method, and (iv) predicting steroidal and non-steroidal AIs. Establishing the ELM model for predicting steroidal and non-steroidal AIs was very simple by replacing the compound data of steroidal AIs with those of non-steroidal AIs without significantly modifying the architecture of the QSAR model.

#### Selecting informative molecular descriptors

The identification of informative molecular descriptors provides an accurate and non-overfitting predictive QSAR model while also providing deeper insight into steroidal and non-steroidal AIs of the aromatase inhibitor (Nantasenamat et al., 2009[17]; Saeys et al., 2007[24]; Shoombuatong et al., 2012[27]). 

Herein, GA was used to select important molecular descriptors (Scrucca, 2012[26]). The foundations of GA were originally developed by Holland (1992[11]) and were based on the evolutionary processes of biological organisms in nature. In this study, selection of informative molecular descriptors was performed such that the ELM model was established through the fitness function of the Akaike information criterion (AIC) and *t*-test (set at a *p*-value < 0.001). The compound was first encoded as molecular descriptors into 637-dimensional and 905-dimensional vectors for steroidal and non-steroidal AIs, respectively, as directly obtained from the previous study (Nantasenamat et al., 2013[19]). Our proposed method offers an easy way to rank and identify informative molecular descriptors using the usage frequency. In this study, the probability of the population size was set at 100 (Scrucca, 2012[26]). Thus, molecular descriptors with 100 and 0 feature usages are the best and worst descriptors of importance, respectively. Finally, a descriptor having high feature usages was then used as a set of informative descriptors to construct the ELM model.

#### Calculating the initial parameter

The proposed method ELM for predicting a chemical compound *C* of aromatase inhibitor was simply formulated by establishing with a weighted summation *f(C)* which was similar to a linear model, as calculated by:


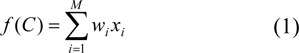


where *w_i _*the *i^th^* parameter and *x_i _*is a *M*-dimensional vector of molecular descriptor. After obtaining descriptor importance, an initial parameter *w_i _*of each selected de-scriptor was calculated by minimizing the sum of squares (ESS) or residual sum of squares (RSS) between the actual and the predicted values as defined:


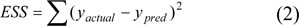


Mathematically, the approximation formula of *w_i_* was given by:


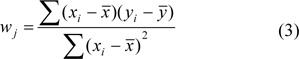


where *y_i_* is a labeled class, *x̅* and *y̅* are the mean value of *x_1_,…,x_N_* and *y_1_,…,y_N_*, respectively, and *N* is a number of compounds.

#### Estimating the optimal parameter

As ELM model constructed using initial parameters could not be guaranteed to afford an efficient QSAR model, therefore, it is desirable for initial parameters W = *w_1_,…,w_M_* to be optimized using a genetic algorithm. Mathematically, the parameter of W = *w_1_,…,w_M_* was obtained from


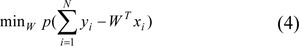


or


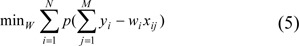


where *p(x) = x^2^* is the sum of squares. In this study, the Andrews' sine function *fitness(x)* (Andrews, 1974[1]; Chatterjee et al., 1996[7]) was applied to estimate the optimal parameter. Practically, the priority of W = *w_1_,…,w_M_* as ranked according to the fitness values that were calculated based on the fitness function *p(x)*. By maximizing the fitness values generation by generation, the optimum parameter with the highest fitness values could be found in the terminal process. To perform the proposed ELM method, the probability of mutation in the parent chromosome, the population size, and the maximum number of generations were set as 0.03, 100, and 1000, respectively (Scrucca, 2012[26]).

#### Prediction of steroidal and non-steroidal AIs

For predicting the aromatase inhibitory activity of a chemical compound *C*, the prediction results (*Pred(C)*) were obtained using the weighted summation *f(C)* and consequently discriminated using only the threshold, as obtained from 


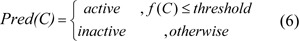


where the threshold was obtained by subtracting the average total weighted summation in the inactive class from the average of total weighted summation in the active class. Because active and inactive classes were encoded with 1 and 2, respectively, a compound with a low-weighted summation *f(C)* tended to be an active class.

### Performance evaluation

Four measurements were used to assess the performance of our proposed ELM method, namely accuracy (Acc), sensitivity (Sen), specificity (Spec), and the Matthews correlation coefficient (MCC) defined as Sen=TP/(TP+FN)*100, Spec=TN/(TN+FP) *100 and Acc=((TP+TN)/(TP+FN+TN+FP)) *100, where TP, TN, FP and *FN* are the number of true positives, true negatives, false positives and false negatives, respectively. The MCC parameter is used in machine learning for evaluating a computational method's performance in binary classification (Vihinen, 2012[30]). The performance of ELM was evaluated using a 10-fold cross-validation (10-fold CV) procedure. For the 10-fold CV process, a dataset was randomly split into ten subsets of roughly the same size. During the experiment with the 10-fold CV, nine of the ten subsets were used for training, and the remaining subset was used for validation. This is carried out iteratively and the final results were averaged across the 10 validated subsets.

## Results and Discussion

In this study, we propose a simple and general-purpose learning method for predicting active and inactive steroidal and non-steroidal AIs. The ELM method was further used in selecting informative molecular descriptors owing to its built-in function for descriptor importance estimation. Due to the non-deterministic characteristics of the ELM method, 10 individual experiments were used to optimize the ELM model. The ELM method was benchmarked with previously reported approach (Nantasenamat et al., 2013[19]). Furthermore, well-known learning methods, i.e., support vector machine (SVM) and artificial neural network (ANN), are also used for comparison with the proposed ELM method. Finally, important molecular descriptors derived from the ELM method were analyzed to further gain insights into the molecular basis of the aromatase inhibitors.

### Prediction accuracy of steroidal AIs

**Figure 2:**
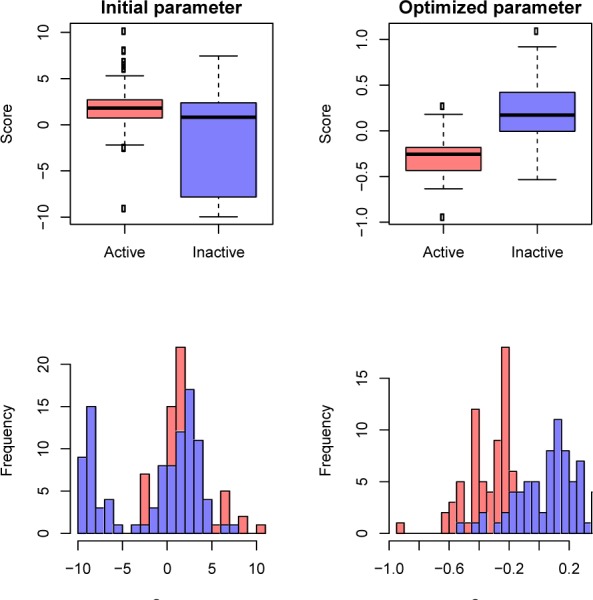
Box and histogram plots of the weighted summation *f(C)* of steroidal AIs obtained using the initial parameter (left) and the optimal parameter (right).

Informative molecular descriptors are critical for designing an accurate QSAR model and providing a good understanding of the aromatase inhibitory activity (Nantasenamat et al., 2009[17], 2010[18]; Saeys et al., 2007[24]; Shoombuatong et al., 2012[27]). After descriptor selection, the list of selected molecular descriptors for constructing the ELM model is shown in Supplementary Table S1. The performance of ELM as a function of parameter optimization can be seen from the histogram and box plot (Figure 2(Fig. 2)) in which the distribution of the weighted summation *f(C)* between the initial (left) and optimized (right) parameters are shown. As observed, the box plot shows that the distribution of *f(C)* using the optimized parameter was well separable compared to using the initial parameter. Furthermore, the histogram clearly shows the decrease of *f(C)* in the overlapping region when using the optimal parameter. It can be assumed that the ELM method could provide an improvement in the performance after optimizing the initial parameter.

The performance of the ELM method using the initial parameter afforded 67.78 % accuracy, 90.12 % sensitivity, 49.49 % specificity, and 0.42 MCC. The QSAR model of ELM using the initial parameter is given below: 


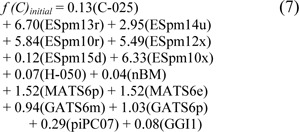


where the threshold was denoted as -0.883. After optimization, the prediction results from 10 individual experiments of ELM using the 10 sets of optimized parameters are given in Table 3(Tab. 3). As observed, the 8^th^ experiment yielded the best performance on the full training data with 85.00 % accuracy, 92.59 % sensitivity, 78.79 % specificity, and 0.71 MCC, and the average result of those 10 individual experiments was in the range of 83.83 ± 0.76 % accuracy, 89.88 ± 1.82 % sensitivity, 78.89 ± 1.54 % specificity, and 0.69 ± 0.02 MCC. As for the result of the 10-fold CV procedure, the threshold of -0.025 in the 7^th^ experiment showed superiority in predicting steroidal AIs by achieving the highest performance of 81.67 % accuracy, 88.89 % sensitivity, 75.76 % specificity, and 0.64 MCC. Meanwhile, the average result of those 10 individual experiments was 80.83 ± 0.71 % accuracy, 87.78 ± 1.82 % sensitivity, 75.15 ± 2.90 % specificity, and 0.63 ± 0.01 MCC. The efficient QSAR model derived from ELM using the optimized parameter is given below: 


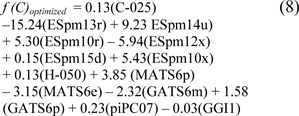


The prediction results of other experiments and their optimal parameter sets are given in Table 3(Tab. 3) and Supplementary Table S1, respectively. As observed from Table 3(Tab. 3), the prediction results from the full training data and the 10-fold CV procedure were not obviously different. These results indicate that our proposed ELM method could alleviate the overfitting problem.

### Prediction accuracy of non-steroidal AIs

**Figure 3:**
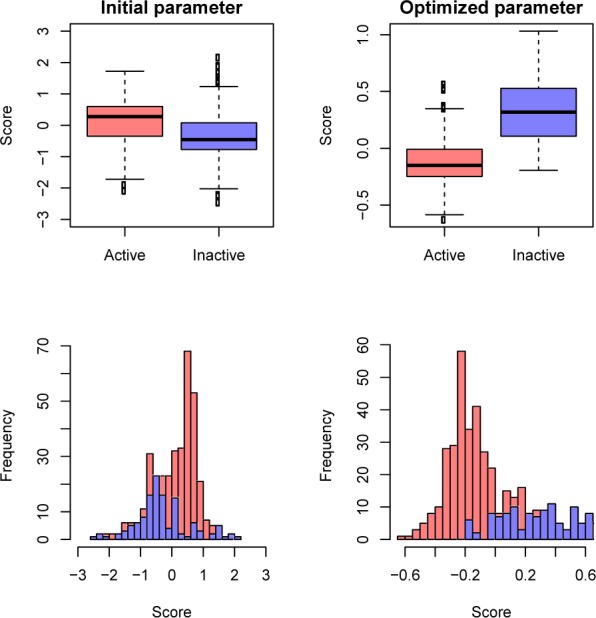
Box and histogram plots of the weighted summation f(c) of non-steroidal AIs obtained using the initial parameter (left) and the optimal parameter (right).

In this study, the establishment of the ELM model for predicting non-steroidal AIs was very simple by replacing the compound of steroidal AIs with non-steroidal AIs. The 15 important molecular descriptors were selected for designing an accurate ELM model, as shown in Supplementary Table S2. In the same way as in the analysis of steroidal AIs, the distribution of the prediction results based on the weighted summation *f(C)* (removed from Eq. 2) based on the weighted summation *f(C)* (removed from Eq. 2) using the initial and optimized parameters are represented with a histogram and box plot, as shown in Figure 3(Fig. 3). The overview distribution between the active and inactive compounds showed that the distribution of *f(C)* was well separable and mitigated the overlapping region after the initial parameter was optimized using the ELM method. This result demonstrates the ability of the ELM method to provide an efficient parameter.

The performance of the proposed ELM method construction with the initial parameter was 61.81 % accuracy, 71.35 % sensitivity, 35.20 % specificity, and 0.06 MCC. The QSAR model based on the ELM method using the initial parameter is given below:


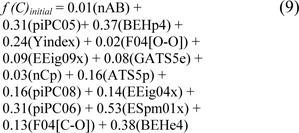


where the threshold was set at -0.912. Meanwhile, Table 4(Tab. 4) shows the prediction results of 10 individual experiments of ELM using the 10 sets of optimized parameters. After the optimization process, the 2^nd^ experiment provided the optimum performance of the full training data with 81.22 % accuracy, 83.95 % sensitivity, 73.60 % specificity, and 0.55 MCC, and the average results of those individual experiments were in the range of 80.70 ± 0.29 % accuracy, 83.58 ± 0.49 % sensitivity, 72.64 ± 1.59 % specificity, and 0.53 ± 0.01 MCC. For the performance of the ELM method with the 10-fold CV procedure, the 4^th^ experiment showed superiority in predicting non-steroidal AIs with a threshold of 0.104. The highest performance was 81.43 % accuracy, 83.67 % sensitivity, 75.20 % specificity, and 0.56 MCC. The average results of accuracy, sensitivity, specificity, and MCC were 80.76 ± 0.33 %, 83.38 ± 0.51 %, 73.44 ± 1.30 %, and 0.54 ± 0.01, respectively. The QSAR model based on the ELM model using the optimized parameter is given below: 


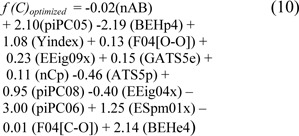


Table 4(Tab. 4) and Supplementary Table S2 provide details of other experiments and their optimized parameter sets, respectively. As observed in Table 4(Tab. 4), the overall prediction results from the full training data and the 10-fold CV procedure were not only obviously different but also performed well in the 10-fold CV procedure.

### Performance of ELM versus the existing and related QSAR methods

A large QSAR model of steroidal and non-steroidal AIs was first proposed by us (Nantasenamat et al., 2013[19]). The decision tree based on the 13 molecular descriptors was applied to discriminate between the active and inactive steroidal and non-steroidal AIs. The prediction results of steroidal AIs yielded as high as 92.22 % accuracy, 93.81 % sensitivity, and 90.36 % specificity when using the full training dataset of AIs, and the results of the 10-fold CV procedure dramatically decreased to 71.67 % accuracy, 76.09 % the ELM method as given in Supplementary Table S1 (7^th^ experiment) and S2 (4^th^ experiment) for steroidal and non-steroidal AIs, respectively. The comparison accuracies of the full training dataset and the 10-fold CV procedure were not very different, ranging from 86.11 % to 81.11 % and from 85.44 % to 78. % for predicting steroidal and non-steroidal AIs, respectively. These results demonstrate that the QSAR model that was established from our selected descriptors afforded significant improvements (Nantasenamat et al., 2013[19]). Furthermore, the well-known learning methods comprising of SVM and ANN were also used to compare with our proposed ELM method. For fair comparisons, the SVM and ANN model were constructed with our selected molecular descriptors and tuned with their optimum parameter. In this study, the SVM model with a radial basis kernel function exp(-γ||*x^i^* - *x^j^*||^2^) from LIBSVM (Chang and Lin, 2011[6]) was used, where *x^i^* and *x^j^* are the *i^th^ and j^th^* compounds of aromatase inhibitors, and γ is a kernel parameter. The parameters y


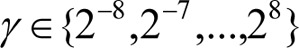


and the cost parameter


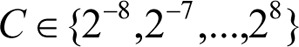


were determined using the grid search method. Meanwhile, the ANN model was optimized by estimating the number of hidden layers (Kuhn, 2008[12]). The comparison results are shown in Table 5(Tab. 5). 

As observed, the QSAR model based on SVM using *C* = 4.0 and γ = 0.0625 provided the best accuracy, specificity, and MCC, which were as high as 82.22 %, 80.81 %, and 0.64, respectively, as evaluated with the 10-fold CV procedure for predicting the activity of steroidal AIs. Meanwhile, the proposed ELM method could afford a comparable prediction results with 81.67 % accuracy, 75.76 % specificity, and 0.64 MCC and also yielded a greater sensitivity. For predicting the activity of non-steroidal AIs, the QSAR model based on SVM using C = 32.0 and γ = 0.0312 and that of our proposed ELM method were comparable and provided higher performances than other QSAR models. The QSAR model based on SVM yielded the highest accuracy and sensitivity at 82.91 % and 93.70 %, respectively, and the ELM method achieved a specificity and MCC of 75.20 % and 0.56, respectively. 

These results indicated that the proposed ELM method could address the following three characteristics: 1) achieve a simple model with acceptable performance at the specified threshold, 2) providing a built-in feature importance estimator and 3) preventing and alleviating the overfitting problem.

### Analysis of important molecular descriptors

**Figure 4:**
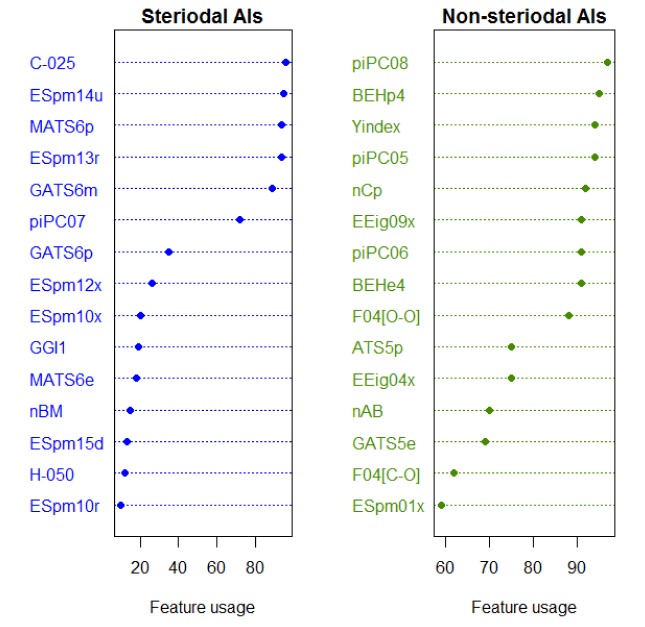
Important molecular descriptors for steroidal (left) and non-steroidal AIs (right), which are ranked according to their feature usages

Molecular descriptors play an important role in improving the QSAR model and providing the essential information of a molecule in terms of its physicochemical properties (Nantasenamat et al., 2009[17]). Thus, the identification of informative molecular descriptors will provide insight into the underlying mechanism of aromatase inhibitors. In this study, a molecular descriptor with the largest feature usage was deemed to be the most efficient descriptor. Figure 4(Fig. 4) shows the value of the feature usage: steroidal (left) and non-steroidal (right) AIs. The top-four informative molecular descriptors of steroidal AIs were C-025, ESpm14u, ESpm13r, and MATS6p, with usage values that were greater than 90. The most important molecular descriptor was C-025, with a feature usage value of 96. Interestingly, 8 out of 15 informative molecular descriptors of non-steroidal AIs had usage values that were greater than 90, and the most important molecular descriptor was piPC08, with a feature usage value of 96. The definition of an informative molecular descriptor is provided in Table 6(Tab. 6). The steroidal and non-steroidal AIs exerted their inhibitory activity via a distinct mechanism. Steroidal AIs competitively and covalently bind the active site of the aromatase enzyme in an irreversible manner (Brueggemeier et al., 1990[4]), whereas non-steroidal AIs coordinates with the heme iron (Fe) atom of the enzyme thereby giving rise to reversible inhibition (Graves and Salhanick, 1979[10]). For the steroidal type, atom-centered-fragments, edge adjacency indices and 2D autocorrelation descriptors were highlighted as informative descriptors with large usage values. C-025 is the most informative one and is defined by looking at the central carbon atom on an aromatic ring and its neighboring atoms. The edge adjacency indices descriptors, i.e., ESpm14u and ESpm13r, represent the connectivity or bonding relationships between the atoms, and MATS6p is involved with the polarizability of molecules. Polarizability is the permanent or induced distortion of electron distribution within a molecule (Nogrady and Weaver, 2005[20]), in other words, the ability of a molecule to be polarized. The presence and/or arrangement of the central carbon atom and its neighbors as well as the bonding relationships between atoms may indicate the size and shape of the compounds, whereas the polarizability is closely related to the hydrophobicity of molecules, and their relationship was noted to be an influencing factor of biological activities (Cammarata, 1967[5]; Leo et al., 1969[13]). For the non-steroidal type, most of the informative molecular descriptors are related with the molecular graph, polarizability, electronegativity of the compound and a certain functional group, i.e., terminal primary C(sp3). The molecular graph represents the structural formula of the compound and may thereby indicate its size and shape. Therefore, it could be hypothesized from our findings that the suitable shape and polarizability of a compound may be essential for both steroidal and non-steroidal AIs in interacting at the enzyme active site and may govern the process of cell entry in reaching the target site of action. In addition, the roles of certain functional groups were noted for the non-steroidal type.

## Conclusion

Computational approaches for predicting steroidal and non-steroidal AIs can accelerate the drug discovery effort and can potentially save cost and time. The continual increase in breast cancer prevalence drives the search for novel aromatase inhibitors. This study proposes the ELM method for the prediction of aromatase inhibitory activity of steroidal and non-steroidal AIs as well as the estimation of its feature importance. This novel algorithm provides a user-friendly QSAR modeling approach with robust predictive performance. Informative molecular descriptors, which were revealed by the feature usage, provided a better understanding on the mechanism of action for the investigated compounds. Our findings suggested that the shape and polarizability of compounds may govern the inhibitory activity of both steroidal and non-steroidal types, whereas the terminal primary C(sp3) functional group and electronegativity may only be required for the non-steroidal type.

## Conflict of interests

The authors have declared that no competing interests exist.

## Acknowledgements

We gratefully acknowledge financial support from the following agencies: Mahidol University Postdoctoral Fellowship Program (to W.S. under supervisions of V.P. and C.N.), Mahidol University Goal-Oriented Research Grant (to C.N.) and the Office of the Higher Education Commission and Mahidol University under the National Research Universities Initiative.

## Supplementary Material

Supplementary material

## Figures and Tables

**Table 1:**
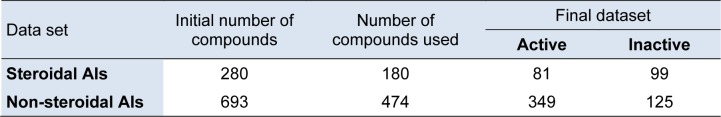
Dataset of steroidal and non-steroidal AIs

**Table 2:**
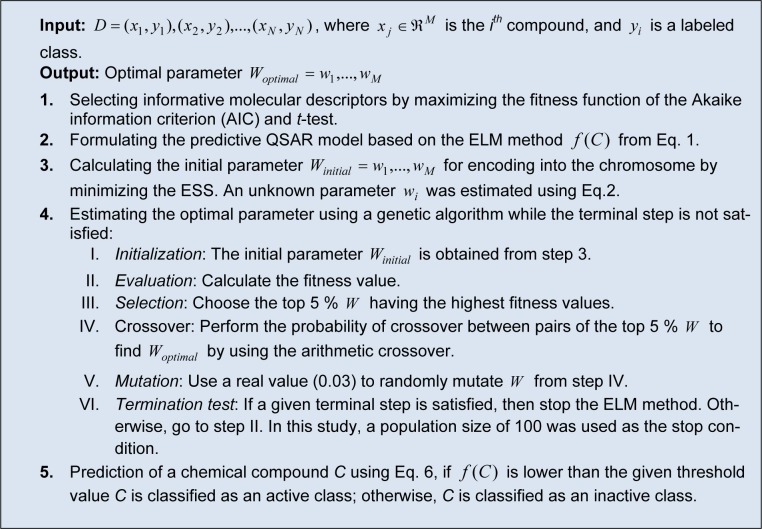
Pseudocode of ELM

**Table 3:**
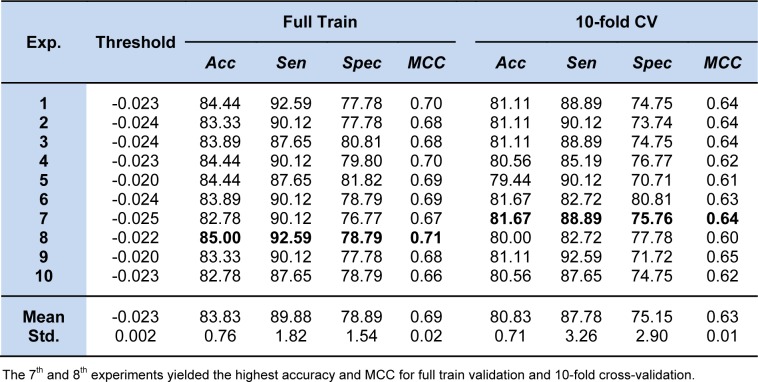
The 10 independent experiments of our proposed ELM method for predicting steroidal AIs

**Table 4:**
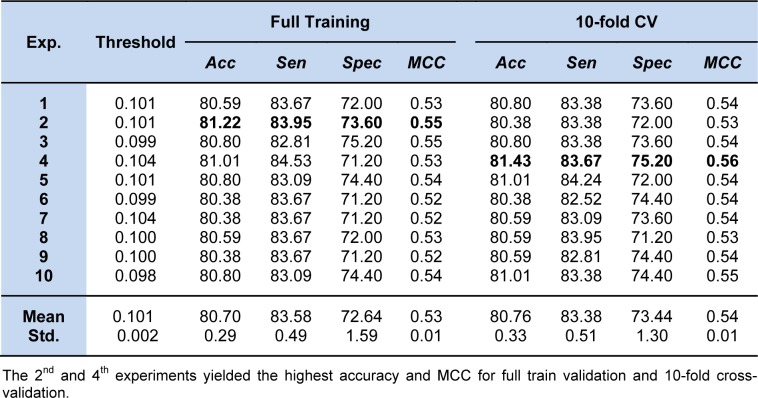
The 10 independent experiments of our proposed ELM method for predicting non-steroidal AIs

**Table 5:**
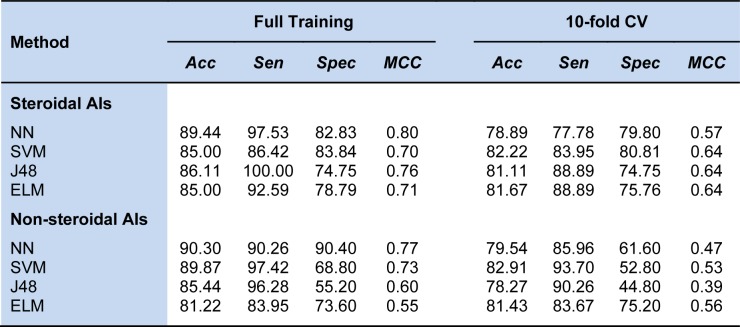
Performance comparison of the proposed ELM method with existing and other related methods

**Table 6:**
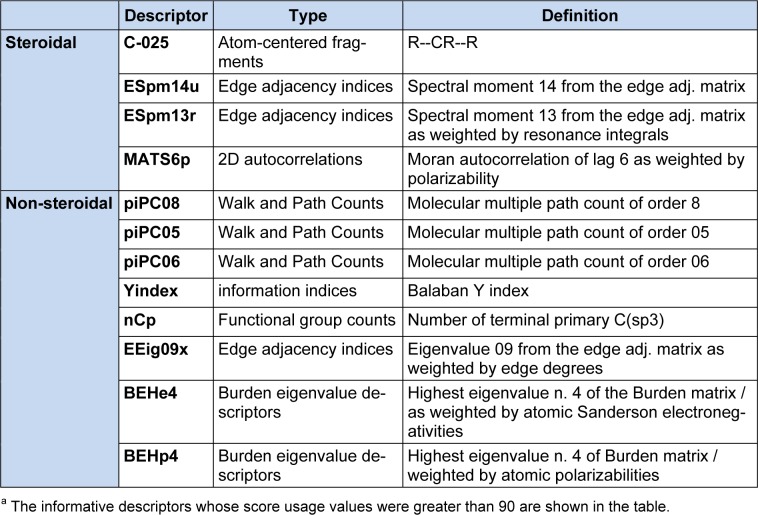
Definition of informative molecular descriptors^a^ of steroidal and non-steroidal AIs
