# Supplementary material for: Prediction of aromatase inhibitory activity using the efficient linear method (ELM)
Source: EXCLI J. 2015 Mar 20;14:452–64. doi: 10.17179/excli2015-140 (PMC4614109; doi:10.17179/excli2015-140)
Supplement: Supplementary material [file EXCLI-14-452-s-001.pdf]

EXCLI Journal 2015;14:452-464 – ISSN 1611-2156
Received: January 10, 2015, accepted: February 02, 2015, published: March 20, 2015

Supplementary material to:

PREDICTION OF AROMATASE INHIBITORY ACTIVITY USING THE EFFICIENT LINEAR METHOD (ELM)

Watshara Shoombuatong[1], Veda Prachayasittikul[1,2], Virapong Prachayasittikul[2], Chanin Nantasenamat[1,2,*]

[1] Center of Data Mining and Biomedical Informatics, Faculty of Medical Technology, Mahidol University, Bangkok 10700, Thailand
[2] Department of Clinical Microbiology and Applied Technology, Faculty of Medical Technology, Mahidol University, Bangkok 10700, Thailand

* Corresponding author: E-mail: chanin.nan@mahidol.ac.th (C.N.); Phone: +66 2 441 4371; Fax: +66 2 441 4380

http://dx.doi.org/10.17179/excli2015-140

This is an Open Access article distributed under the terms of the Creative Commons Attribution License (http://creativecommons.org/licenses/by/4.0/).

Supplementary Table S1: Initial and 10 optimal parameter sets of ELM for predicting steroidal AIs

Feature	Initial	1	2	3	4	5	6	7*	8	9	10
C-025	0.13	-0.21	0.08	0.36	0.19	0.19	0.17	0.13	0.11	0.33	-0.06
ESpm13r	6.70	-13.48	-7.93	-15.55	-11.25	-11.25	-9.18	-15.24	-11.07	-9.62	-9.77
ESpm14u	2.95	7.30	7.12	11.57	6.33	6.33	9.18	9.23	9.71	8.05	4.53
ESpm10r	5.84	2.57	-0.90	5.65	4.32	4.32	2.34	5.30	3.99	0.00	5.23
ESpm12x	5.49	-0.54	-2.72	-6.68	-6.27	-6.27	-8.32	-5.94	-3.09	-8.67	-3.46
ESpm15d	0.12	0.13	0.09	0.21	0.12	0.12	0.19	0.15	0.13	0.32	0.26
ESpm10x	6.33	1.01	2.12	3.74	6.76	6.76	3.79	5.43	-2.69	9.73	1.85
H-050	0.07	0.03	0.05	0.06	0.09	0.09	0.05	0.13	0.03	0.07	0.09
nBM	0.04	0.03	0.00	-0.02	-0.03	-0.03	0.03	0.00	0.10	-0.04	0.03
MATS6p	1.52	3.93	3.18	3.88	3.34	3.34	3.84	3.85	2.37	3.08	4.48
MATS6e	1.52	-2.46	-2.44	-2.63	-1.88	-1.88	-3.24	-3.15	-1.80	-2.29	-4.02
GATS6m	0.94	-2.44	-1.92	-2.27	-1.69	-1.69	-1.99	-2.32	-1.48	-2.12	-2.58
GATS6p	1.03	2.62	1.35	2.41	1.89	1.89	1.40	1.58	1.01	1.86	1.72
piPC07	0.29	0.62	0.64	0.74	0.26	0.26	0.72	0.23	0.46	0.02	0.41
GGI1	0.08	0.02	-0.01	-0.02	-0.02	-0.02	-0.06	-0.03	0.03	-0.11	-0.09

* Experiment 7 provided the highest performance for predicting steroidal AIs

EXCLI Journal 2015;14:452-464 – ISSN 1611-2156
Received: January 10, 2015, accepted: February 02, 2015, published: March 20, 2015

Supplementary Table S2: Initial and 10 optimal parameter sets of ELM for predicting non-steroidal AIs

Feature	Initial	1	2	3	4*	5	6	7	8	9	10
**nAB**	0.01	-0.02	-0.03	-0.02	-0.02	-0.02	-0.03	-0.02	-0.02	-0.02	-0.02
**piPC05**	0.31	2.17	1.94	1.83	2.10	1.81	2.16	2.21	1.92	2.06	1.96
**BEHp4**	0.37	-1.92	-2.30	-2.23	-2.19	-2.01	-1.97	-1.83	-1.93	-2.18	-1.79
**Yindex**	0.24	0.88	1.00	0.96	1.08	0.95	0.96	0.96	1.06	1.04	1.09
**F04[O-O]**	0.02	0.13	0.11	0.12	0.13	0.13	0.11	0.13	0.12	0.12	0.11
**EEig09x**	0.09	0.25	0.20	0.22	0.23	0.21	0.21	0.22	0.21	0.24	0.19
**GATS5e**	0.08	0.17	0.17	0.14	0.15	0.12	0.13	0.13	0.13	0.15	0.11
**nCp**	0.03	0.11	0.10	0.10	0.11	0.11	0.11	0.14	0.10	0.10	0.12
**ATS5p**	0.16	-0.51	-0.43	-0.41	-0.46	-0.46	-0.48	-0.48	-0.54	-0.49	-0.54
**piPC08**	0.16	0.90	0.94	1.05	0.95	1.03	0.89	0.98	0.95	1.03	0.90
**EEig04x**	0.14	-0.37	-0.51	-0.33	-0.40	-0.34	-0.37	-0.35	-0.49	-0.30	-0.35
**piPC06**	0.31	-2.92	-2.88	-2.97	-3.00	-2.92	-2.85	-3.11	-2.85	-3.02	-2.79
**ESpm01x**	0.53	1.06	1.88	1.24	1.25	1.22	1.06	1.24	1.68	1.03	1.33
**F04[C-O]**	0.01	-0.01	-0.01	-0.01	-0.01	-0.01	-0.01	-0.01	-0.01	-0.01	-0.01
**BEHe4**	0.38	1.89	2.05	2.15	2.14	1.94	2.05	1.66	1.92	2.21	1.73

* Experiment 4 provided the highest performance for predicting non-steroidal AIs
